# Non-technical skills progression during anesthesiology residency in Portugal: the impact of a National Pedagogical Plan

**DOI:** 10.1080/10872981.2020.1800980

**Published:** 2020-08-20

**Authors:** Francisco Maio Matos, Mafalda Ramos Martins, Inês Martins

**Affiliations:** a Faculdade de Ciência da Saúde, Universidade da Beira Interior, Covilhã, Portugal; b Department of Anesthesiology and Biomedical Simulation Centre, Centro Hospitalar e Universitário de Coimbra, Coimbra, Portugal

**Keywords:** Anesthesiology, simulation, behavior, residency, pedagogical plan, self-assessment, non-technical skills

## Abstract

Background

Simulation is known as an important tool for the learning of technical and non-technical skills without endangering patient safety. In Portugal, a National Pedagogical Plan for Anesthesiology Residents was created based on simulation training. This plan was designed according to the objectives set forth by the Portuguese Board of Anesthesiology. This study aimed to evaluate the impact of simulation training courses on the non-technical skills of medical residents in Anesthesiology.

Methods

Confidential questionnaires, pre- and post-course, were answered by all the residents that attended the different modules of the simulation training program at Centro Hospitalar e Universitário de Coimbra Biomedical Simulation Centre, Portugal, from February 2011 to March 2018.

Results

A total of 344 questionnaires were answered. In the group of questions regarding the need for help, mistakes, and self-efficacy over time, students recognized an increase over time in the need for support and the self-assessment of the number of mistakes (p < 0.001). Regarding the self-evaluation of safety culture and communication skills, at the end of the residency, almost all the students recognized that they did not feel bad when asking for help or expressing their opinion, even when they disagreed with the consultant anesthesiologist. This was significantly different from the values of the self-assessment at the beginning of residency (p < 0.001). The evolution of preparation, knowledge, and training also showed a positive evolution over the simulation modules (p < 0.001). Finally, the evaluation of the behavioral component in the clinical setting showed a significant positive evolution over time (p < 0.001): in the end, all the students strongly agreed that behavioral competencies are crucial.

Conclusions

The impact of simulation on anesthesiology non-technical skills during residency is positive and recognized by the students. Moreover, simulation also helps in the recognition of error, enriching the value of self-confidence and the crucial role of behavioral skills.

ABBREVIATIONS

BSC-CHUC: Biomedical Simulation Centre from Centro Hospitalar e Universitário de Coimbra

## Background

In medical education, students need to acquire the necessary skills to treat and care for patients. Due to the complexity of patient care, namely in the field of anesthesiology, where clinicians have to face with emergencies and multidisciplinary teams, knowledge is not limited to technical procedures but also include a behavioral component such as the ability to communicate with other healthcare providers or patients, teamwork, situation awareness, and decision making [1,2]. Thus, the main objectives of medical education include not only the acquisition of theoretical knowledge with scientific evidence, technical and non-technical, but also behavioral competencies [3]. During medical education, students are supposed to comprehend their clinical self-evolution, increase their awareness of error and their gaps, and develop their behavioral skills [4].

Simulation could replace real experiences, in an immersive and interactive environment, allowing participants to learn and acquire skills in a controlled way with the guarantee of patient safety [5]. With simulation, real patients are replaced by artificial models, live actors, or virtual reality patients, aiming to replicate patient care scenarios in a realistic environment [6]. Due to the impact in the learning course and in retention time, medical simulation allows for an improvement of this process [7]. Moreover, simulation will contribute to filling technical and non-technical lacunae, both belonging to the process of clinical evolution [6].

Although some reports state that simulation can enhance critical thinking and behavior, data on how these skills can be transferred to real patients is lacking, and therefore more research in this field is needed [8].

The development of a National Pedagogic Plan by the Biomedical Simulation Centre from Centro Hospitalar e Universitário de Coimbra (BSC-CHUC) in Portugal has the objective of integrating a simulation-based training for Anesthesiology residents, as part of their training, including non-technical skills.

This study aimed to evaluate how a simulation program applied to Portuguese Anesthesiology residents, over the four years of residency, could impact the acquisition of behavioral competencies by the students. This evaluation was performed with a new instrument, a questionnaire, that was created to self-assess aspects of anesthetic practice that would be difficult to evaluate by direct observation, such as the ability to manage a crisis.

Data were collected using confidential questionnaires given before and after each simulation module corresponding to the specific year of residency, including individual and team learning, behavior, and course evaluation questions. Thus, we will be able to identify gaps in knowledge and practice that are fundamental motivators to continuing professional development. The evidence suggests that improved accuracy of self-assessment leads to improved learning outcomes. This may be relevant to the emerging field of simulation-based learning [9].

In this paper data related to behavior will be presented. This belongs to a Kirkpatrick level 2 since we will be able to demonstrate that simulation changed the performance outside of the clinical environment [2].

## Methods

### Study design

This was a prospective observational study designed to evaluate how the Anesthesiology Simulation Pedagogical Plan from BSC-CHUC impacted the behavior of Portuguese Anesthesiology residents. To achieve this goal, questionnaires were applied before and after each simulation module. These questionnaires were designed according to the pedagogical contents of each year of the Anesthesiology Residency Program (ARP) [10,11].

Questionnaires included questions regarding learning, behavior, and evaluation of the pedagogical content of each simulation course. Behavioral questions were similar throughout the four years (horizontal questionnaire – Table 1) and were performed before and after each simulation module. The complete questionnaires are included in Supplementary Data (Additional File 1).

**Table 1.** Horizontal questionnaire applied over the 4 years of the ARP. These questions were performed pre- and post-simulation courses each year.

	Question
**Q5**	I have been in situations that I could not deal with without help
**Q6**	I ask for help
**Q7**	I feel the need for support
**Q8**	I make mistakes
**Q9**	It is difficult for me to report the mistakes I make
**Q10**	I do not feel prepared for the responsibility I have
**Q11**	I do not have enough knowledge for the responsibility I have
**Q12**	I do not have enough training for the responsibility I have
**Q13**	I do not have enough experience for the responsibility I have
**Q14**	I feel bad when I ask for help
**Q15**	When I disagree with the consultant anesthesiologist’s opinion, I do not express that position
**Q16**	The behavioral component is crucial in the clinical setting

### Questionnaires development and validation

The draft questionnaire was designed by two anesthesiologists with experience in simulation. To ensure face and content validity, the items were reviewed for syntax and appropriateness by a panel of 5 experts with expertise in the area of simulation in anesthesiology training. The final questions were evaluated by a behavioral psychologist for the rejection of confounder items [12].

The questionnaires were administered to 30 participants of the Anesthesiology Simulation Pedagogical Plan from BSC-CHUC, in two pilot-courses. These participants were anesthesiology residents from CHUC belonging to the target group of the questionnaires.

Internal reliability was estimated for the overall questionnaires using Cronbach´s alpha coefficient. The values obtained for each year’s questionnaire were: 0.86 for year I, 0.84 for year II, 0.87 for year III, and 0.89 for year IV, indicating high internal consistency of all questionnaires.

### Setting and participants

This observational study was conducted in Portugal, from 2011 to 2018, at BSC-CHUC. The participants were Anesthesiology residents that attended the simulation courses at BSC-CHUC. Inclusion criteria: all residents enrolled in anesthesiology simulation courses at BSC-CHUC.

### Variables and methods of assessment

All variables were collected on an anonymized database specifically designed for the study. The source of all variables were the specific questionnaires applied before and after each simulation course. Answers to Q5, Q6, Q7, and Q8 were given on a three-point Likert Scale (0-never; 1-few times; 2-many times) and the remaining on a five-point Likert Scale (0-strongly disagree; 1-partially disagree; 2-no opinion; 3-partially agree; 4-strongly agree).

### Bias

Not applicable.

### Quantitative variables

All collected variables were quantitative.

### Statistical methods

Non-parametric statistical methods were used. All analyses were performed with the Wilcoxon test. Values are presented as mean (95% confidence intervals). SPSSv20 (IBM, USA) was used. Tests were considered significant at α < 0.05 significance level (two-sided).

## Results

A total of 344 answered questionnaires were included in the study. The first-year course was concluded by 76 residents, the second year by 89, the third year by 82, and the fourth year by 93 residents. The mean age of the residents, in the first year, was 26.5 years with a minimum of 25 years and a maximum of 29 years.

**Figure 1.**
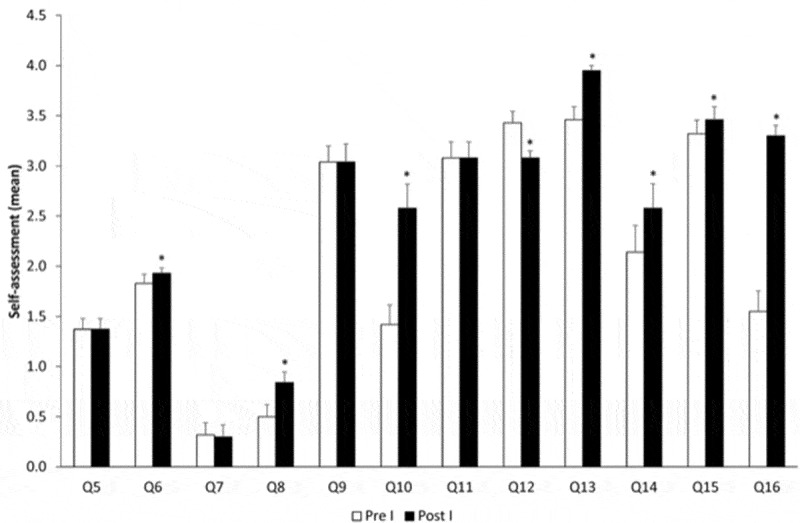
Evolution over time comparing pre-course and post-course – year I. *p < 0.05.

**Figure 2.**
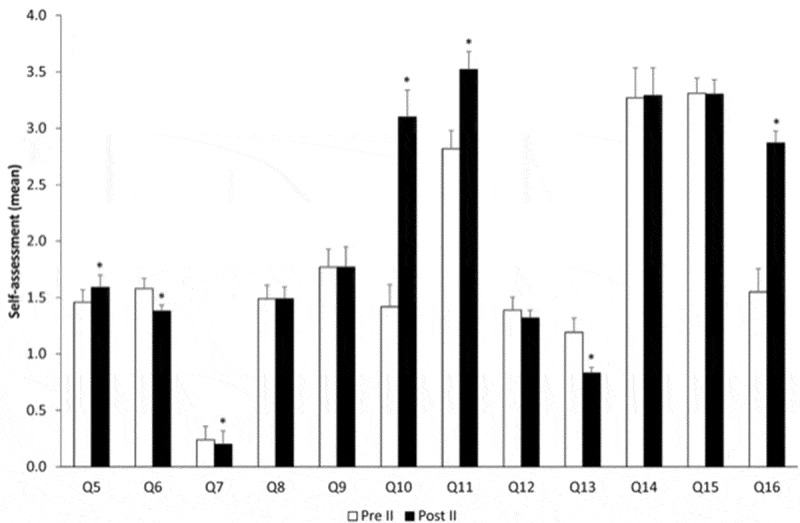
Evolution over time comparing pre-course and post-course – year II. *p < 0.05.

**Figure 3.**
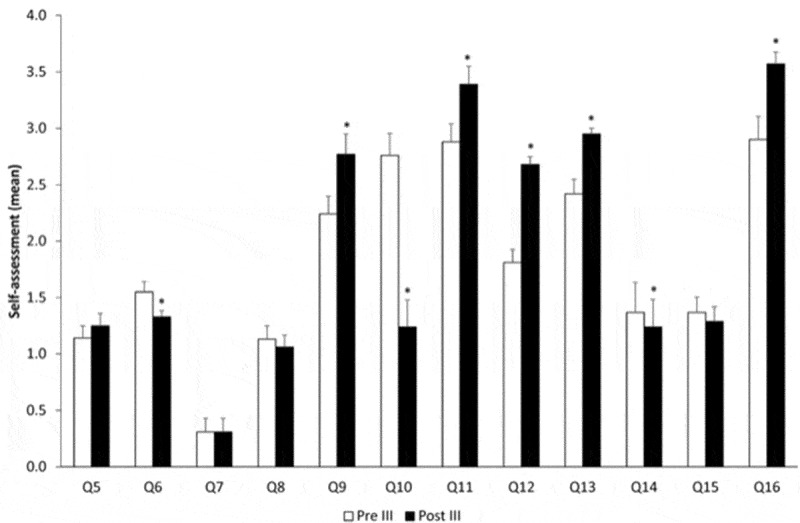
Evolution over time comparing pre-course and post-course – year III. *p < 0.05.

**Figure 4.**
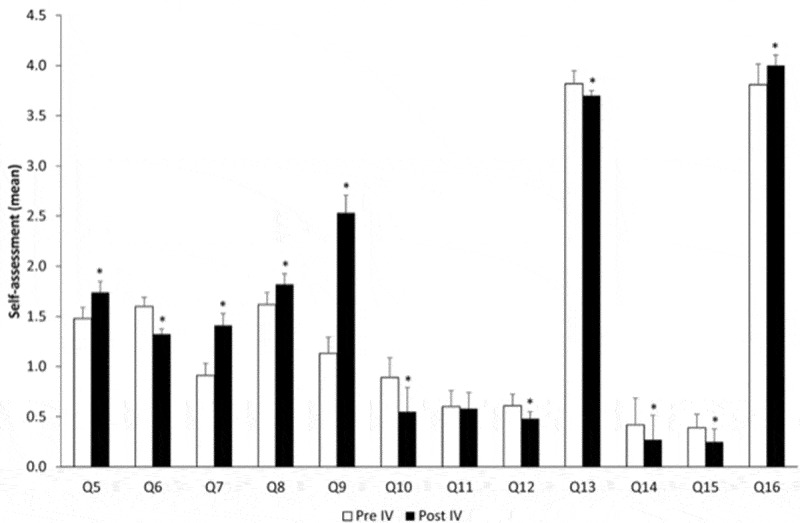
Evolution over time comparing pre-course and post-course – year IV. *p < 0.05.

Figures 1–4 represent the self-assessment of the residents before and after each simulation course in the first, second, third, and fourth year, respectively.

In the first year (Figure 1), the simulation course allowed the residents to gain more confidence to ask for help (Q6); they acknowledged they made more mistakes (Q8), and they felt less prepared and with less experience towards their responsibility (Q10 and Q13). Students felt an increase in the training for their responsibility (Q12) and more students assumed to feel bad when asking for help (14). Nevertheless, they were more confident to share their opinion (Q15) and attributed more importance to the behavioral component (Q16).

After the second-year course (Figure 2), students recognized to face more situations that they could not deal with without help (Q5). Nevertheless, they asked for help less often (Q6) and also felt less need for support (Q7). Regarding their responsibility, after the simulation course, they felt that they were less prepared (Q10), did not have enough knowledge (Q11) but had more experience (Q13). Similarly to the first-year course, the behavioral component gained more importance after the simulation course (Q16).

The third year of the simulation course provided the anesthesiology residents with the ability to ask for help less (Q6). However, they felt difficulty to report the mistakes they made (Q9). Although feeling more prepared for their responsibility (Q10), the students acknowledged that they had less knowledge, less training, and less experience (Q11, Q12, and Q13). The course decreased the fact that they felt bad when asking for help (Q14). The behavioral component gained even more importance after this third-course year (Q16).

The last simulation course was the one that impacted more on the students’ self-evaluation. There was only one answer that was not changed with the course (Q11, regarding the knowledge for responsibility).

**Figure 5.**
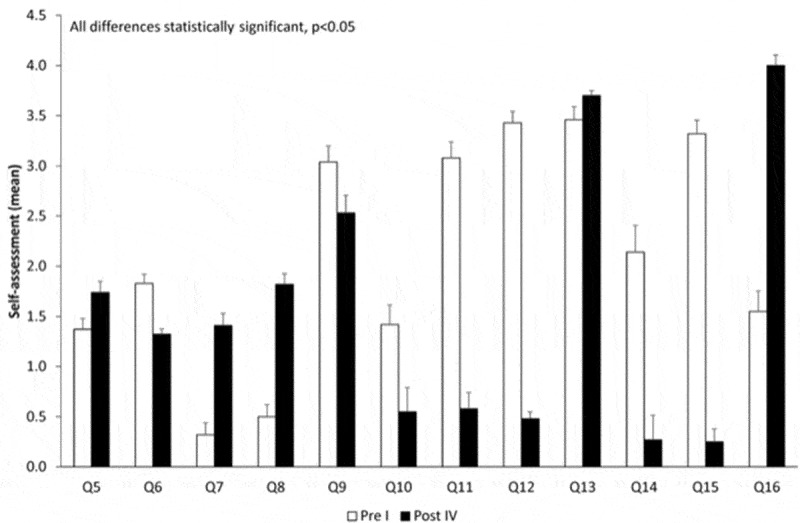
Global evolution of the simulation courses. All differences are statistically significant.

Figure 5 represents the global evolution from the pre-year I course to post-year IV course. All differences are statistically significant.

## Discussion

Medical error is an important cause of death and some errors are attributed to skills such as communication and leadership [13,14]. Simulation activities, beyond their known impact on the learning of technical skills in a safe environment, are a powerful form of concrete and active experiences with a high retention level that potentially changes behaviors (leadership, communication, and resource management), ultimately increasing patient safety [6,15,16]. Therefore, they can be considered a bridge between theoretical lessons and clinical practice, helping junior doctors to deal with emergencies [17].

However, the optimal use of simulation in anesthesiology education programs is not so clear [2]. In our National Pedagogic Plan from the BSC-CHUC, a simulation course was proposed to all Portuguese residents in anesthesiology, designed according to the curricular goals set forth by the Portuguese College of Anesthesiology and had a wide participation showing the interest of residents in the study.

Using questionnaires, we aimed to contribute to better understand the role and importance of simulation in non-technical skills that are fundamental for the correct and safe practice in anesthesiology.

Taken together our results showed that the simulation courses positively impact the learning process of the students.

The ‘ask for help’ (Q6) was one of the questions whose answer changed after every simulation course. Nevertheless, only in the first year there was an increased self-perception regarding the need for help. This suggests that the first year allows the students to gain conscientiousness about their limitations. Another important point to underline is the increase in the ‘need for support’ (Q7) in the fourth year. Indeed, the fourth year was the one where students felt more need for support (Q7), suggesting that this feeling increased after being exposed several times to simulation scenarios.

Regarding Q8, it was very interesting to observe that first-year residents were the ones that perceived to make fewer mistakes, either because they still did not have the opportunity to do them or because they did not have enough knowledge to realize them. On the contrary, the 4th year was the most experienced and knowledgeable, and when students recognized to make more mistakes. This suggests that the more we know the more we can self-criticize. Also, experience and autonomy increased in the later years and this increased the likelihood of acknowledging errors. Notwithstanding, only during the third and fourth years, students felt more difficulty to report their own mistakes (Q9).

A common point that may explain the answers to these questions is the fact that first-year students are always accompanied, and therefore feel more comfortable. In the second year, students gain more awareness of their limitations due to the increase in experience and knowledge. In the third year, the know-how and the confidence increase considerably. Finally, in the last year, the fourth, they face critical situations that they are not able to solve on their own and gain consciousness regarding errors and, consequently, the need for help.

In the questions focusing on the residents’ perception about responsibility, it is important to underline that only after the fourth year the students acknowledged to be more prepared (Q10), more trained (Q12), and to have more experience (Q13) after the simulation training. The positive impact on knowledge occurred after the second and third years (Q11). The preparedness acknowledged by the residents in the later years is very important and critical situations during the simulation courses contributed to this preparedness. In the third-year students have to face situations with more responsibility and in a more autonomous manner. Also, in the third year, the level of difficulty increases, and therefore students need to go through an adaptation process. If we interpret the results regarding experience based on simulation training, we may speculate that students felt that they needed more simulation training during the residency.

Regarding communication skills, the main differences were the increase in the ‘ask for help’ (Q14) and opinion expression (Q15) after the 1st course, with opposite results in the last year: decrease of both. These results suggest that students acquired more confidence in themselves, recognizing both a higher need to ask for help and a higher confidence to express their opinion to the consultant anesthesiologist over the years. These results can be related to the fact that students felt more familiar with the department, more confident, more patient, more aware of what matters – they were not as concerned as before regarding what others may think about them. As the residency progresses, students understand that asking for help is a basic component of the general clinical practice and specifically of anesthesiology. Communication skills are fundamental and impact numerous health outcomes, including trust in clinicians, satisfaction, and even patient and family quality of life [18–20]. Therefore, training in communication skills is crucial to improve them [21]. Our results show that the simulation courses positively impact the communication skills of anesthesiology residents. The simulation scenario also helps students to understand that the leader is not necessarily the older person but the one that better faces and solves specific situations, at specific moments. Everyone´s opinion is a valuable input for every situation, and a lack of leadership could be highly detrimental to performance during a critical situation [22].

Finally, the global question about behavior in critical situations clearly showed that although the increase during the first simulation course is a significant behavioral component, it gained more importance throughout the residency. One possible explanation may be that at the beginning of training there was a strong focus on lack of knowledge and skills, and less focus in behavior. Also, behavior was less tested in the first years since at that time residents are not alone and do not make clinical decisions. Given this implies less leadership, non-technical skills were not considered important [22]. However, and over the years, students had no doubts about the impact of behavior in critical situations, which is clearly reflected on the fourth-year being the one that attributed more importance to behavioral questions. This year was the one with the most experience and that faced a higher number of situations that showed them the importance of non-technical skills. Also, they have had the opportunity to participate in courses and congresses that have enhanced these characteristics, and therefore are better able to understand these skills as crucial in the clinical setting.

Approaches including teamwork, mistakes, communication, and need for help have been considered a priority in the simulation setting since they can have an impact on patient safety. Therefore, including these approaches in simulation will allow to identify latent threats in a clinical environment [16,23].

Following the success of this program, a broader project was developed, under the coordination of the Portuguese Medical Association. This project aimed to create a national training program recommended to Anesthesiology residents and was designed in cooperation with all Portuguese simulation centers.

The main limitation of this study is the fact that it was only based on students´ self-evaluation. Therefore, the results of the simulation training were only presented from the students’ point of view. An independent evaluation should be performed to validate the results from other points of view. Another limitation is the fact that only residents that voluntarily enrolled in the program were included: it was not randomized and that could have influenced the results since the participants can be more prone and willing to learn. Finally, the simulated environment could not fully capture the real behavior that would occur in a real environment. However, this limitation is inherent to all simulation training.

## Conclusion

This study shows that a simulation program positively impacts non-technical/behavioral issues, influencing the learning process in Anesthesiology, corresponding to a Kirkpatrick level 2. Further studies will be performed to confirm the ability to recognize the crucial importance of non-technical skills in the clinical setting.

## Supplementary Material

Supplemental Material

## Data Availability

The datasets used and/or analyzed during the current study are available from the corresponding author on reasonable request.
