# Supplementary material for: Non-technical skills progression during anesthesiology residency in Portugal: the impact of a National Pedagogical Plan
Source: Med Educ Online. 2020 Aug 20;25(1):1800980. doi: 10.1080/10872981.2020.1800980 (PMC7482781; doi:10.1080/10872981.2020.1800980)
Supplement: Supplemental Material [file ZMEO_A_1800980_SM9828.docx]

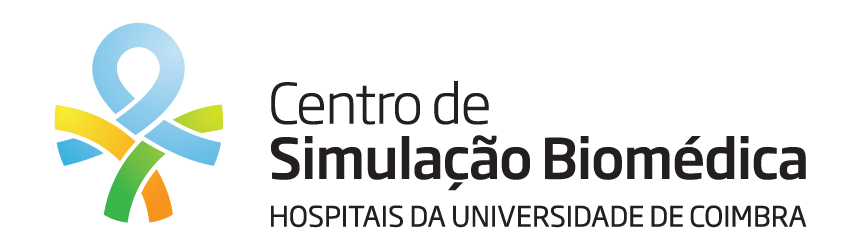


Choose a number:

1, 2, 3, 4, 5, 6, 7, 8, 9, 10, 11, 12, 13, 14, 15, 16, 17, 18, 19, 20

Questionnaire – Module I

Dear colleague,

This questionnaire intends to assess the evolution of the participant during the training with simulation. The expected average time for completion is five minutes. All data is confidential and used for research purposes only.

The medical team of Coimbra’s Biomedical Simulation Center be grateful for your cooperation.

Their fill shall be understood as an authorization to the assumptions referred above.

1. How do you assess your preparation for critical events in the operating room or emergency room?

| Null |  |  |  |  |  |  |  |  |  | Maximum |
| --- | --- | --- | --- | --- | --- | --- | --- | --- | --- | --- |
| 0 | 1 | 2 | 3 | 4 | 5 | 6 | 7 | 8 | 9 | 10 |

1. In your opinion, how important is…

2.1… airway managment?

| Null |  |  |  |  |  |  |  |  |  | Maximum |
| --- | --- | --- | --- | --- | --- | --- | --- | --- | --- | --- |
| 0 | 1 | 2 | 3 | 4 | 5 | 6 | 7 | 8 | 9 | 10 |

2.2. ventilatory monitoring?

| Null |  |  |  |  |  |  |  |  |  | Maximum |
| --- | --- | --- | --- | --- | --- | --- | --- | --- | --- | --- |
| 0 | 1 | 2 | 3 | 4 | 5 | 6 | 7 | 8 | 9 | 10 |

2.3.. cardiac monitoring?

| Null |  |  |  |  |  |  |  |  |  | Maximum |
| --- | --- | --- | --- | --- | --- | --- | --- | --- | --- | --- |
| 0 | 1 | 2 | 3 | 4 | 5 | 6 | 7 | 8 | 9 | 10 |

2.4… neuromuscular block monitoring?

| Null |  |  |  |  |  |  |  |  |  | Maximum |
| --- | --- | --- | --- | --- | --- | --- | --- | --- | --- | --- |
| 0 | 1 | 2 | 3 | 4 | 5 | 6 | 7 | 8 | 9 | 10 |

1. How do you evaluate your training ...

3.1… in difficult airway management?

| Null |  |  |  |  |  |  |  |  |  | Maximum |
| --- | --- | --- | --- | --- | --- | --- | --- | --- | --- | --- |
| 0 | 1 | 2 | 3 | 4 | 5 | 6 | 7 | 8 | 9 | 10 |

3.2… in advanced life support?

| Null |  |  |  |  |  |  |  |  |  | Maximum |
| --- | --- | --- | --- | --- | --- | --- | --- | --- | --- | --- |
| 0 | 1 | 2 | 3 | 4 | 5 | 6 | 7 | 8 | 9 | 10 |

3.3… for emergencies for emergencies in your clinical practice?

| Null |  |  |  |  |  |  |  |  |  | Maximum |
| --- | --- | --- | --- | --- | --- | --- | --- | --- | --- | --- |
| 0 | 1 | 2 | 3 | 4 | 5 | 6 | 7 | 8 | 9 | 10 |

3.4… in crisis resource management?

| Null |  |  |  |  |  |  |  |  |  | Maximum |
| --- | --- | --- | --- | --- | --- | --- | --- | --- | --- | --- |
| 0 | 1 | 2 | 3 | 4 | 5 | 6 | 7 | 8 | 9 | 10 |

1. How do you rate your expertise…

4.1… for difficult airway management?

| Null |  |  |  |  |  |  |  |  |  | Maximum |
| --- | --- | --- | --- | --- | --- | --- | --- | --- | --- | --- |
| 0 | 1 | 2 | 3 | 4 | 5 | 6 | 7 | 8 | 9 | 10 |

4.2… in advanced life support?

| Null |  |  |  |  |  |  |  |  |  | Maximum |
| --- | --- | --- | --- | --- | --- | --- | --- | --- | --- | --- |
| 0 | 1 | 2 | 3 | 4 | 5 | 6 | 7 | 8 | 9 | 10 |

4.3… for emergencies in your clinical practice?

| Null |  |  |  |  |  |  |  |  |  | Maximum |
| --- | --- | --- | --- | --- | --- | --- | --- | --- | --- | --- |
| 0 | 1 | 2 | 3 | 4 | 5 | 6 | 7 | 8 | 9 | 10 |

4.4… in crisis resource management?

| Null |  |  |  |  |  |  |  |  |  | Maximum |
| --- | --- | --- | --- | --- | --- | --- | --- | --- | --- | --- |
| 0 | 1 | 2 | 3 | 4 | 5 | 6 | 7 | 8 | 9 | 10 |

1. I've been in situations that I couldn’t deal without help.

| Never | Few times | Many times |
| --- | --- | --- |
| 🞎 | 🞎 | 🞎 |

1. I call for help

| Never | Few times | Many times |
| --- | --- | --- |
| 🞎 | 🞎 | 🞎 |

1. I feel the need for support

| Never | Few times | Many times |
| --- | --- | --- |
| 🞎 | 🞎 | 🞎 |

1. I make mistakes

| Never | Few times | Many times |
| --- | --- | --- |
| 🞎 | 🞎 | 🞎 |

1. It’s difficult for me to report the mistakes I make

| Strongly disagree | Partially disagree | No opinion | Partially agree | Strongly agree |
| --- | --- | --- | --- | --- |
| 🞎 | 🞎 | 🞎 | 🞎 | 🞎 |

1. I don’t feel prepared for the responsibility I have

| Strongly disagree | Partially disagree | No opinion | Partially agree | Strongly agree |
| --- | --- | --- | --- | --- |
| 🞎 | 🞎 | 🞎 | 🞎 | 🞎 |

1. I don’t have enough knowledge for the responsibility I have

| Strongly disagree | Partially disagree | No opinion | Partially agree | Strongly agree |
| --- | --- | --- | --- | --- |
| 🞎 | 🞎 | 🞎 | 🞎 | 🞎 |

1. I don’t have enough training for the responsibility I have

| Strongly disagree | Partially disagree | No opinion | Partially agree | Strongly agree |
| --- | --- | --- | --- | --- |
| 🞎 | 🞎 | 🞎 | 🞎 | 🞎 |

1. I don’t have enough experience for the responsibility I have

| Strongly disagree | Partially disagree | No opinion | Partially agree | Strongly agree |
| --- | --- | --- | --- | --- |
| 🞎 | 🞎 | 🞎 | 🞎 | 🞎 |

1. I feel bad when I call for help

| Strongly disagree | Partially disagree | No opinion | Partially agree | Strongly agree |
| --- | --- | --- | --- | --- |
| 🞎 | 🞎 | 🞎 | 🞎 | 🞎 |

1. When I disagree with the consultant anesthesiologist opinion, I don’t express that position.

| Strongly disagree | Partially disagree | No opinion | Partially agree | Strongly agree |
| --- | --- | --- | --- | --- |
| 🞎 | 🞎 | 🞎 | 🞎 | 🞎 |

1. The behavioural component is crucial in the clinical setting

| Strongly disagree | Partially disagree | No opinion | Partially agree | Strongly agree |
| --- | --- | --- | --- | --- |
| 🞎 | 🞎 | 🞎 | 🞎 | 🞎 |

1. Simulation team training is an important complement to the residency program

| Strongly disagree | Partially disagree | No opinion | Partially agree | Strongly agree |
| --- | --- | --- | --- | --- |
| 🞎 | 🞎 | 🞎 | 🞎 | 🞎 |

1. A regular simulation update plan should be defined

| Strongly disagree | Partially disagree | No opinion | Partially agree | Strongly agree |
| --- | --- | --- | --- | --- |
| 🞎 | 🞎 | 🞎 | 🞎 | 🞎 |

1. Simulation team training improves clinical daily practice

| Strongly disagree | Partially disagree | No opinion | Partially agree | Strongly agree |
| --- | --- | --- | --- | --- |
| 🞎 | 🞎 | 🞎 | 🞎 | 🞎 |

1. Simulation team training have an impact on patients' clinical outcome

| Strongly disagree | Partially disagree | No opinion | Partially agree | Strongly agree |
| --- | --- | --- | --- | --- |
| 🞎 | 🞎 | 🞎 | 🞎 | 🞎 |

Course evaluation

Pedagogical content

1. Basic Pharmacology in Anesthesiology

Subject importance

| Null |  |  |  |  |  |  |  |  |  | Maximum |
| --- | --- | --- | --- | --- | --- | --- | --- | --- | --- | --- |
| 0 | 1 | 2 | 3 | 4 | 5 | 6 | 7 | 8 | 9 | 10 |

1. Basic and advanced airway

Subject importance

| Null |  |  |  |  |  |  |  |  |  | Maximum |
| --- | --- | --- | --- | --- | --- | --- | --- | --- | --- | --- |
| 0 | 1 | 2 | 3 | 4 | 5 | 6 | 7 | 8 | 9 | 10 |

1. Ventilation

Subject importance

| Null |  |  |  |  |  |  |  |  |  | Maximum |
| --- | --- | --- | --- | --- | --- | --- | --- | --- | --- | --- |
| 0 | 1 | 2 | 3 | 4 | 5 | 6 | 7 | 8 | 9 | 10 |

1. Vascular cannulation in anesthesia

Subject importance

| Null |  |  |  |  |  |  |  |  |  | Maximum |
| --- | --- | --- | --- | --- | --- | --- | --- | --- | --- | --- |
| 0 | 1 | 2 | 3 | 4 | 5 | 6 | 7 | 8 | 9 | 10 |

1. Ultrasound in anesthesia

Subject importance

| Null |  |  |  |  |  |  |  |  |  | Maximum |
| --- | --- | --- | --- | --- | --- | --- | --- | --- | --- | --- |
| 0 | 1 | 2 | 3 | 4 | 5 | 6 | 7 | 8 | 9 | 10 |

1. Central and peripheral cannulation using ultrasound

Subject importance

| Null |  |  |  |  |  |  |  |  |  | Maximum |
| --- | --- | --- | --- | --- | --- | --- | --- | --- | --- | --- |
| 0 | 1 | 2 | 3 | 4 | 5 | 6 | 7 | 8 | 9 | 10 |

1. Neuroaxial anesthesia and local anesthetics

Subject importance

| Null |  |  |  |  |  |  |  |  |  | Maximum |
| --- | --- | --- | --- | --- | --- | --- | --- | --- | --- | --- |
| 0 | 1 | 2 | 3 | 4 | 5 | 6 | 7 | 8 | 9 | 10 |

1. Simulation training on operation room - clinical cases

28.1Subject importance

| Null |  |  |  |  |  |  |  |  |  | Maximum |
| --- | --- | --- | --- | --- | --- | --- | --- | --- | --- | --- |
| 0 | 1 | 2 | 3 | 4 | 5 | 6 | 7 | 8 | 9 | 10 |

28.2Formative impact

| Null |  |  |  |  |  |  |  |  |  | Maximum |
| --- | --- | --- | --- | --- | --- | --- | --- | --- | --- | --- |
| 0 | 1 | 2 | 3 | 4 | 5 | 6 | 7 | 8 | 9 | 10 |

1. Simulation training on post-anesthesia care unit - clinical cases

29.1Subject importance

| Null |  |  |  |  |  |  |  |  |  | Maximum |
| --- | --- | --- | --- | --- | --- | --- | --- | --- | --- | --- |
| 0 | 1 | 2 | 3 | 4 | 5 | 6 | 7 | 8 | 9 | 10 |

29.2Formative impact

| Null |  |  |  |  |  |  |  |  |  | Maximum |
| --- | --- | --- | --- | --- | --- | --- | --- | --- | --- | --- |
| 0 | 1 | 2 | 3 | 4 | 5 | 6 | 7 | 8 | 9 | 10 |

1. Etiology and prevention of cardio-respiratory arrest

Subject importance

| Null |  |  |  |  |  |  |  |  |  | Maximum |
| --- | --- | --- | --- | --- | --- | --- | --- | --- | --- | --- |
| 0 | 1 | 2 | 3 | 4 | 5 | 6 | 7 | 8 | 9 | 10 |

1. Basic Life Support (BLS) algorithm

Subject importance

| Null |  |  |  |  |  |  |  |  |  | Maximum |
| --- | --- | --- | --- | --- | --- | --- | --- | --- | --- | --- |
| 0 | 1 | 2 | 3 | 4 | 5 | 6 | 7 | 8 | 9 | 10 |

1. Advanced Life Support (ALS) algorithm

Subject importance

| Null |  |  |  |  |  |  |  |  |  | Maximum |
| --- | --- | --- | --- | --- | --- | --- | --- | --- | --- | --- |
| 0 | 1 | 2 | 3 | 4 | 5 | 6 | 7 | 8 | 9 | 10 |

1. Recognition of rhythms

Subject importance

| Null |  |  |  |  |  |  |  |  |  | Maximum |
| --- | --- | --- | --- | --- | --- | --- | --- | --- | --- | --- |
| 0 | 1 | 2 | 3 | 4 | 5 | 6 | 7 | 8 | 9 | 10 |

1. Defibrillation

Subject importance

| Null |  |  |  |  |  |  |  |  |  | Maximum |
| --- | --- | --- | --- | --- | --- | --- | --- | --- | --- | --- |
| 0 | 1 | 2 | 3 | 4 | 5 | 6 | 7 | 8 | 9 | 10 |

1. Simulation training on ALS - clinical cases

35.1 Subject importance

| Null |  |  |  |  |  |  |  |  |  | Maximum |
| --- | --- | --- | --- | --- | --- | --- | --- | --- | --- | --- |
| 0 | 1 | 2 | 3 | 4 | 5 | 6 | 7 | 8 | 9 | 10 |

35.2 Formative impact

| Null |  |  |  |  |  |  |  |  |  | Maximum |
| --- | --- | --- | --- | --- | --- | --- | --- | --- | --- | --- |
| 0 | 1 | 2 | 3 | 4 | 5 | 6 | 7 | 8 | 9 | 10 |

1. Global evaluation

| Null |  |  |  |  |  |  |  |  |  | Maximum |
| --- | --- | --- | --- | --- | --- | --- | --- | --- | --- | --- |
| 0 | 1 | 2 | 3 | 4 | 5 | 6 | 7 | 8 | 9 | 10 |


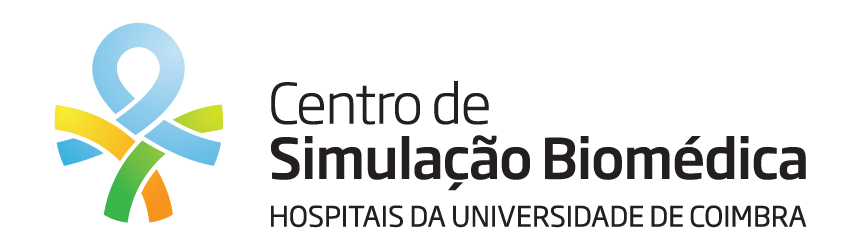
Choose a letter:

| **A** | **B** | **C** | **D** | **E** | **F** | **G** | **H** | **I** | **J** | **L** | **M** | **N** | **O** | **P** | **Q** | **R** | **S** | **T** |
| --- | --- | --- | --- | --- | --- | --- | --- | --- | --- | --- | --- | --- | --- | --- | --- | --- | --- | --- |

Questionnaire – Module II

Dear colleague,

This questionnaire intends to assess the evolution of the participant during the training with simulation. The expected average time for completion is five minutes.

All data is confidential and used for research purposes only.

The medical team of Coimbra’s Biomedical Simulation Center be grateful for your cooperation.

Their fill shall be understood as an authorization to the assumptions referred above.

1. How do you assess your preparation for critical events in the operating room or emergency room?

| Null |  |  |  |  |  |  |  |  |  | Maximum |
| --- | --- | --- | --- | --- | --- | --- | --- | --- | --- | --- |
| 0 | 1 | 2 | 3 | 4 | 5 | 6 | 7 | 8 | 9 | 10 |

1. In your opinion, how important is…

2.1… airway management?

| Null |  |  |  |  |  |  |  |  |  | Maximum |
| --- | --- | --- | --- | --- | --- | --- | --- | --- | --- | --- |
| 0 | 1 | 2 | 3 | 4 | 5 | 6 | 7 | 8 | 9 | 10 |

2.2... ventilatory monitoring?

| Null |  |  |  |  |  |  |  |  |  | Maximum |
| --- | --- | --- | --- | --- | --- | --- | --- | --- | --- | --- |
| 0 | 1 | 2 | 3 | 4 | 5 | 6 | 7 | 8 | 9 | 10 |

2.3... cardiac monitoring?

| Null |  |  |  |  |  |  |  |  |  | Maximum |
| --- | --- | --- | --- | --- | --- | --- | --- | --- | --- | --- |
| 0 | 1 | 2 | 3 | 4 | 5 | 6 | 7 | 8 | 9 | 10 |

2.4… neuromuscular block monitoring?

| Null |  |  |  |  |  |  |  |  |  | Maximum |
| --- | --- | --- | --- | --- | --- | --- | --- | --- | --- | --- |
| 0 | 1 | 2 | 3 | 4 | 5 | 6 | 7 | 8 | 9 | 10 |

1. How do you evaluate your training ...

3.1… in difficult airway management?

| Null |  |  |  |  |  |  |  |  |  | Maximum |
| --- | --- | --- | --- | --- | --- | --- | --- | --- | --- | --- |
| 0 | 1 | 2 | 3 | 4 | 5 | 6 | 7 | 8 | 9 | 10 |

3.2… in advanced life support?

| Null |  |  |  |  |  |  |  |  |  | Maximum |
| --- | --- | --- | --- | --- | --- | --- | --- | --- | --- | --- |
| 0 | 1 | 2 | 3 | 4 | 5 | 6 | 7 | 8 | 9 | 10 |

3.3… for emergencies for emergencies in your clinical practice?

| Null |  |  |  |  |  |  |  |  |  | Maximum |
| --- | --- | --- | --- | --- | --- | --- | --- | --- | --- | --- |
| 0 | 1 | 2 | 3 | 4 | 5 | 6 | 7 | 8 | 9 | 10 |

3.4… in crisis resource management?

| Null |  |  |  |  |  |  |  |  |  | Maximum |
| --- | --- | --- | --- | --- | --- | --- | --- | --- | --- | --- |
| 0 | 1 | 2 | 3 | 4 | 5 | 6 | 7 | 8 | 9 | 10 |

1. How do you rate your expertise…

4.1… for difficult airway management?

| Null |  |  |  |  |  |  |  |  |  | Maximum |
| --- | --- | --- | --- | --- | --- | --- | --- | --- | --- | --- |
| 0 | 1 | 2 | 3 | 4 | 5 | 6 | 7 | 8 | 9 | 10 |

4.2… in advanced life support?

| Null |  |  |  |  |  |  |  |  |  | Maximum |
| --- | --- | --- | --- | --- | --- | --- | --- | --- | --- | --- |
| 0 | 1 | 2 | 3 | 4 | 5 | 6 | 7 | 8 | 9 | 10 |

4.3… for emergencies in your clinical practice?

| Null |  |  |  |  |  |  |  |  |  | Maximum |
| --- | --- | --- | --- | --- | --- | --- | --- | --- | --- | --- |
| 0 | 1 | 2 | 3 | 4 | 5 | 6 | 7 | 8 | 9 | 10 |

4.4… in crisis resource management?

| Null |  |  |  |  |  |  |  |  |  | Maximum |
| --- | --- | --- | --- | --- | --- | --- | --- | --- | --- | --- |
| 0 | 1 | 2 | 3 | 4 | 5 | 6 | 7 | 8 | 9 | 10 |

1. I've been in situations that I couldn’t deal without help.

| Never | Few times | Many times |
| --- | --- | --- |
| 🞎 | 🞎 | 🞎 |

1. I call for help

| Never | Few times | Many times |
| --- | --- | --- |
| 🞎 | 🞎 | 🞎 |

1. I feel the need for support

| Never | Few times | Many times |
| --- | --- | --- |
| 🞎 | 🞎 | 🞎 |

1. I make mistakes

| Never | Few times | Many times |
| --- | --- | --- |
| 🞎 | 🞎 | 🞎 |

1. It’s difficult for me to report the mistakes I make

| Strongly disagree | Partially disagree | No opinion | Partially agree | Strongly agree |
| --- | --- | --- | --- | --- |
| 🞎 | 🞎 | 🞎 | 🞎 | 🞎 |

1. I don’t feel prepared for the responsibility I have

| Strongly disagree | Partially disagree | No opinion | Partially agree | Strongly agree |
| --- | --- | --- | --- | --- |
| 🞎 | 🞎 | 🞎 | 🞎 | 🞎 |

1. I don’t have enough knowledge for the responsibility I have

| Strongly disagree | Partially disagree | No opinion | Partially agree | Strongly agree |
| --- | --- | --- | --- | --- |
| 🞎 | 🞎 | 🞎 | 🞎 | 🞎 |

1. I don’t have enough training for the responsibility I have

| Strongly disagree | Partially disagree | No opinion | Partially agree | Strongly agree |
| --- | --- | --- | --- | --- |
| 🞎 | 🞎 | 🞎 | 🞎 | 🞎 |

1. I don’t have enough experience for the responsibility I have

| Strongly disagree | Partially disagree | No opinion | Partially agree | Strongly agree |
| --- | --- | --- | --- | --- |
| 🞎 | 🞎 | 🞎 | 🞎 | 🞎 |

1. I feel bad when I call for help

| Strongly disagree | Partially disagree | No opinion | Partially agree | Strongly agree |
| --- | --- | --- | --- | --- |
| 🞎 | 🞎 | 🞎 | 🞎 | 🞎 |

1. When I disagree with the consultant anesthesiologist opinion, I don’t express that position.

| Strongly disagree | Partially disagree | No opinion | Partially agree | Strongly agree |
| --- | --- | --- | --- | --- |
| 🞎 | 🞎 | 🞎 | 🞎 | 🞎 |

1. The behavioural component is crucial in the clinical setting

| Strongly disagree | Partially disagree | No opinion | Partially agree | Strongly agree |
| --- | --- | --- | --- | --- |
| 🞎 | 🞎 | 🞎 | 🞎 | 🞎 |

1. Simulation team training is an important complement to the residency program

| Strongly disagree | Partially disagree | No opinion | Partially agree | Strongly agree |
| --- | --- | --- | --- | --- |
| 🞎 | 🞎 | 🞎 | 🞎 | 🞎 |

1. A regular simulation update plan should be defined

| Strongly disagree | Partially disagree | No opinion | Partially agree | Strongly agree |
| --- | --- | --- | --- | --- |
| 🞎 | 🞎 | 🞎 | 🞎 | 🞎 |

1. Simulation team training improves clinical daily practice

| Strongly disagree | Partially disagree | No opinion | Partially agree | Strongly agree |
| --- | --- | --- | --- | --- |
| 🞎 | 🞎 | 🞎 | 🞎 | 🞎 |

1. Simulation team training have an impact on patients' clinical outcome

| Strongly disagree | Partially disagree | No opinion | Partially agree | Strongly agree |
| --- | --- | --- | --- | --- |
| 🞎 | 🞎 | 🞎 | 🞎 | 🞎 |

**Course evaluation**

**Pedagogical content**

1. Leadership and health management

Subject importance

| Null |  |  |  |  |  |  |  |  |  | Maximum |
| --- | --- | --- | --- | --- | --- | --- | --- | --- | --- | --- |
| 0 | 1 | 2 | 3 | 4 | 5 | 6 | 7 | 8 | 9 | 10 |

1. Difficult Airway Algorithm

Subject importance

| Null |  |  |  |  |  |  |  |  |  | Maximum |
| --- | --- | --- | --- | --- | --- | --- | --- | --- | --- | --- |
| 0 | 1 | 2 | 3 | 4 | 5 | 6 | 7 | 8 | 9 | 10 |

1. Supraglottic and transcutaneous devices

Subject importance

| Null |  |  |  |  |  |  |  |  |  | Maximum |
| --- | --- | --- | --- | --- | --- | --- | --- | --- | --- | --- |
| 0 | 1 | 2 | 3 | 4 | 5 | 6 | 7 | 8 | 9 | 10 |

1. Fibroscopy principles

Subject importance

| Null |  |  |  |  |  |  |  |  |  | Maximum |
| --- | --- | --- | --- | --- | --- | --- | --- | --- | --- | --- |
| 0 | 1 | 2 | 3 | 4 | 5 | 6 | 7 | 8 | 9 | 10 |

1. Simulation training on difficult airway - clinical cases

41.1 Subject relevance

| Null |  |  |  |  |  |  |  |  |  | Maximum |
| --- | --- | --- | --- | --- | --- | --- | --- | --- | --- | --- |
| 0 | 1 | 2 | 3 | 4 | 5 | 6 | 7 | 8 | 9 | 10 |

41.2 Formative impact

| Null |  |  |  |  |  |  |  |  |  | Maximum |
| --- | --- | --- | --- | --- | --- | --- | --- | --- | --- | --- |
| 0 | 1 | 2 | 3 | 4 | 5 | 6 | 7 | 8 | 9 | 10 |

1. Ultrasound in anaesthesiology

Subject importance

| Null |  |  |  |  |  |  |  |  |  | Maximum |
| --- | --- | --- | --- | --- | --- | --- | --- | --- | --- | --- |
| 0 | 1 | 2 | 3 | 4 | 5 | 6 | 7 | 8 | 9 | 10 |

1. Ultrasound guided regional blocks

Subject importance

| Null |  |  |  |  |  |  |  |  |  | Maximum |
| --- | --- | --- | --- | --- | --- | --- | --- | --- | --- | --- |
| 0 | 1 | 2 | 3 | 4 | 5 | 6 | 7 | 8 | 9 | 10 |

1. Simulation training on ultrasound guided regional blocks - clinical cases

44.1 Subject relevance

| Null |  |  |  |  |  |  |  |  |  | Maximum |
| --- | --- | --- | --- | --- | --- | --- | --- | --- | --- | --- |
| 0 | 1 | 2 | 3 | 4 | 5 | 6 | 7 | 8 | 9 | 10 |

44.2 Formative impact

| Null |  |  |  |  |  |  |  |  |  | Maximum |
| --- | --- | --- | --- | --- | --- | --- | --- | --- | --- | --- |
| 0 | 1 | 2 | 3 | 4 | 5 | 6 | 7 | 8 | 9 | 10 |

1. Anesthetic approach of the burned patient

Subject importance

| Null |  |  |  |  |  |  |  |  |  | Maximum |
| --- | --- | --- | --- | --- | --- | --- | --- | --- | --- | --- |
| 0 | 1 | 2 | 3 | 4 | 5 | 6 | 7 | 8 | 9 | 10 |

1. Simulation training on the anaesthetic approach to the burned patient - clinical cases

46.1 Subject relevance

| Null |  |  |  |  |  |  |  |  |  |  | Maximum |
| --- | --- | --- | --- | --- | --- | --- | --- | --- | --- | --- | --- |
| 0 |  | 1 | 2 | 3 | 4 | 5 | 6 | 7 | 8 | 9 | 10 |

46.2 Formative impact

| Null |  |  |  |  |  |  |  |  |  | Maximum |
| --- | --- | --- | --- | --- | --- | --- | --- | --- | --- | --- |
| 0 | 1 | 2 | 3 | 4 | 5 | 6 | 7 | 8 | 9 | 10 |

1. Global evaluation

| Null |  |  |  |  |  |  |  |  |  | Maximum |
| --- | --- | --- | --- | --- | --- | --- | --- | --- | --- | --- |
| 0 | 1 | 2 | 3 | 4 | 5 | 6 | 7 | 8 | 9 | 10 |


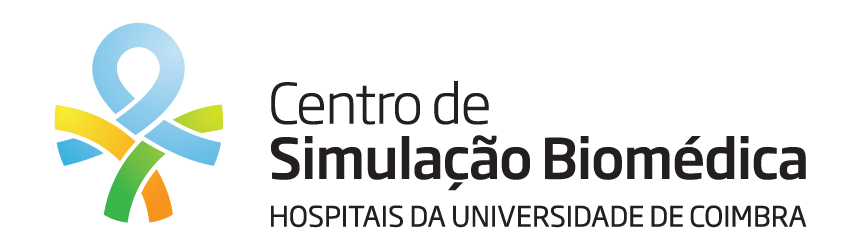
Choose a letter:

| **A** | **B** | **C** | **D** | **E** | **F** | **G** | **H** | **I** | **J** | **L** | **M** | **N** | **O** | **P** | **Q** | **R** | **S** | **T** |
| --- | --- | --- | --- | --- | --- | --- | --- | --- | --- | --- | --- | --- | --- | --- | --- | --- | --- | --- |

Questionnaire – Module III

Dear colleague,

This questionnaire intends to assess the evolution of the participant during the training with simulation. The expected average time for completion is five minutes.

All data is confidential and used for research purposes only.

The medical team of Coimbra’s Biomedical Simulation Center be grateful for your cooperation.

Their fill shall be understood as an authorization to the assumptions referred above.

1. How do you assess your preparation for critical events in the operating room or emergency room?

| Null |  |  |  |  |  |  |  |  |  | Maximum |
| --- | --- | --- | --- | --- | --- | --- | --- | --- | --- | --- |
| 0 | 1 | 2 | 3 | 4 | 5 | 6 | 7 | 8 | 9 | 10 |

1. In your opinion, how important is…

2.1… airway managment?

| Null |  |  |  |  |  |  |  |  |  | Maximum |
| --- | --- | --- | --- | --- | --- | --- | --- | --- | --- | --- |
| 0 | 1 | 2 | 3 | 4 | 5 | 6 | 7 | 8 | 9 | 10 |

2.2... ventilatory monitoring?

| Null |  |  |  |  |  |  |  |  |  | Maximum |
| --- | --- | --- | --- | --- | --- | --- | --- | --- | --- | --- |
| 0 | 1 | 2 | 3 | 4 | 5 | 6 | 7 | 8 | 9 | 10 |

2.3... cardiac monitoring?

| Null |  |  |  |  |  |  |  |  |  | Maximum |
| --- | --- | --- | --- | --- | --- | --- | --- | --- | --- | --- |
| 0 | 1 | 2 | 3 | 4 | 5 | 6 | 7 | 8 | 9 | 10 |

2.4… neuromuscular block monitoring?

| Null |  |  |  |  |  |  |  |  |  | Maximum |
| --- | --- | --- | --- | --- | --- | --- | --- | --- | --- | --- |
| 0 | 1 | 2 | 3 | 4 | 5 | 6 | 7 | 8 | 9 | 10 |

1. How do you evaluate your training...

3.1… in difficult airway management?

| Null |  |  |  |  |  |  |  |  |  | Maximum |
| --- | --- | --- | --- | --- | --- | --- | --- | --- | --- | --- |
| 0 | 1 | 2 | 3 | 4 | 5 | 6 | 7 | 8 | 9 | 10 |

3.2… in advanced life support?

| Null |  |  |  |  |  |  |  |  |  | Maximum |
| --- | --- | --- | --- | --- | --- | --- | --- | --- | --- | --- |
| 0 | 1 | 2 | 3 | 4 | 5 | 6 | 7 | 8 | 9 | 10 |

3.3… for emergencies in your clinical practice?

| Null |  |  |  |  |  |  |  |  |  | Maximum |
| --- | --- | --- | --- | --- | --- | --- | --- | --- | --- | --- |
| 0 | 1 | 2 | 3 | 4 | 5 | 6 | 7 | 8 | 9 | 10 |

3.4… in crisis resource management?

| Null |  |  |  |  |  |  |  |  |  | Maximum |
| --- | --- | --- | --- | --- | --- | --- | --- | --- | --- | --- |
| 0 | 1 | 2 | 3 | 4 | 5 | 6 | 7 | 8 | 9 | 10 |

3.5… in obstetric emergencies?

| Null |  |  |  |  |  |  |  |  |  | Maximum |
| --- | --- | --- | --- | --- | --- | --- | --- | --- | --- | --- |
| 0 | 1 | 2 | 3 | 4 | 5 | 6 | 7 | 8 | 9 | 10 |

3.6… in trauma?

| Null |  |  |  |  |  |  |  |  |  | Maximum |
| --- | --- | --- | --- | --- | --- | --- | --- | --- | --- | --- |
| 0 | 1 | 2 | 3 | 4 | 5 | 6 | 7 | 8 | 9 | 10 |

1. How do you rate your expertise…

4.1… for difficult airway management?

| Null |  |  |  |  |  |  |  |  |  | Maximum |
| --- | --- | --- | --- | --- | --- | --- | --- | --- | --- | --- |
| 0 | 1 | 2 | 3 | 4 | 5 | 6 | 7 | 8 | 9 | 10 |

4.2… in advanced life support?

| Null |  |  |  |  |  |  |  |  |  | Maximum |
| --- | --- | --- | --- | --- | --- | --- | --- | --- | --- | --- |
| 0 | 1 | 2 | 3 | 4 | 5 | 6 | 7 | 8 | 9 | 10 |

4.3… for emergencies in your clinical practice?

| Null |  |  |  |  |  |  |  |  |  | Maximum |
| --- | --- | --- | --- | --- | --- | --- | --- | --- | --- | --- |
| 0 | 1 | 2 | 3 | 4 | 5 | 6 | 7 | 8 | 9 | 10 |

4.4… in crisis resource management?

| Null |  |  |  |  |  |  |  |  |  | Maximum |
| --- | --- | --- | --- | --- | --- | --- | --- | --- | --- | --- |
| 0 | 1 | 2 | 3 | 4 | 5 | 6 | 7 | 8 | 9 | 10 |

4.5… in obstetric emergencies?

| Null |  |  |  |  |  |  |  |  |  | Maximum |
| --- | --- | --- | --- | --- | --- | --- | --- | --- | --- | --- |
| 0 | 1 | 2 | 3 | 4 | 5 | 6 | 7 | 8 | 9 | 10 |

4.6… in trauma?

| Null |  |  |  |  |  |  |  |  |  | Maximum |
| --- | --- | --- | --- | --- | --- | --- | --- | --- | --- | --- |
| 0 | 1 | 2 | 3 | 4 | 5 | 6 | 7 | 8 | 9 | 10 |

1. I've been in situations that I couldn’t deal without help.

| Never | Few times | Many times |
| --- | --- | --- |
| 🞎 | 🞎 | 🞎 |

1. I call for help

| Never | Few times | Many times |
| --- | --- | --- |
| 🞎 | 🞎 | 🞎 |

1. I feel the need for support

| Never | Few times | Many times |
| --- | --- | --- |
| 🞎 | 🞎 | 🞎 |

1. I make mistakes

| Never | Few times | Many times |
| --- | --- | --- |
| 🞎 | 🞎 | 🞎 |

1. It’s difficult for me to report the mistakes I make

| Strongly disagree | Partially disagree | No opinion | Partially agree | Strongly agree |
| --- | --- | --- | --- | --- |
| 🞎 | 🞎 | 🞎 | 🞎 | 🞎 |

1. I don’t feel prepared for the responsibility I have

| Strongly disagree | Partially disagree | No opinion | Partially agree | Strongly agree |
| --- | --- | --- | --- | --- |
| 🞎 | 🞎 | 🞎 | 🞎 | 🞎 |

1. I don’t have enough knowledge for the responsibility I have

| Strongly disagree | Partially disagree | No opinion | Partially agree | Strongly agree |
| --- | --- | --- | --- | --- |
| 🞎 | 🞎 | 🞎 | 🞎 | 🞎 |

1. I don’t have enough training for the responsibility I have

| Strongly disagree | Partially disagree | No opinion | Partially agree | Strongly agree |
| --- | --- | --- | --- | --- |
| 🞎 | 🞎 | 🞎 | 🞎 | 🞎 |

1. I don’t have enough experience for the responsibility I have

| Strongly disagree | Partially disagree | No opinion | Partially agree | Strongly agree |
| --- | --- | --- | --- | --- |
| 🞎 | 🞎 | 🞎 | 🞎 | 🞎 |

1. I feel bad when I call for help

| Strongly disagree | Partially disagree | No opinion | Partially agree | Strongly agree |
| --- | --- | --- | --- | --- |
| 🞎 | 🞎 | 🞎 | 🞎 | 🞎 |

1. When I disagree with the consultant anesthesiologist opinion, I don’t express that position.

| Strongly disagree | Partially disagree | No opinion | Partially agree | Strongly agree |
| --- | --- | --- | --- | --- |
| 🞎 | 🞎 | 🞎 | 🞎 | 🞎 |

1. The behavioural component is crucial in the clinical setting

| Strongly disagree | Partially disagree | No opinion | Partially agree | Strongly agree |
| --- | --- | --- | --- | --- |
| 🞎 | 🞎 | 🞎 | 🞎 | 🞎 |

1. Simulation team training is an important complement to the residency program

| Strongly disagree | Partially disagree | No opinion | Partially agree | Strongly agree |
| --- | --- | --- | --- | --- |
| 🞎 | 🞎 | 🞎 | 🞎 | 🞎 |

1. A regular simulation update plan should be defined

| Strongly disagree | Partially disagree | No opinion | Partially agree | Strongly agree |
| --- | --- | --- | --- | --- |
| 🞎 | 🞎 | 🞎 | 🞎 | 🞎 |

1. Simulation team training improves clinical daily practice

| Strongly disagree | Partially disagree | No opinion | Partially agree | Strongly agree |
| --- | --- | --- | --- | --- |
| 🞎 | 🞎 | 🞎 | 🞎 | 🞎 |

1. Simulation team training have an impact on patients' clinical outcome

| Strongly disagree | Partially disagree | No opinion | Partially agree | Strongly agree |
| --- | --- | --- | --- | --- |
| 🞎 | 🞎 | 🞎 | 🞎 | 🞎 |

**Course evaluation**

**Pedagogical content**

1. Assessment of a trauma patient, head and thoracic trauma

Subject importance

| Null |  |  |  |  |  |  |  |  |  | Maximum |
| --- | --- | --- | --- | --- | --- | --- | --- | --- | --- | --- |
| 0 | 1 | 2 | 3 | 4 | 5 | 6 | 7 | 8 | 9 | 10 |

1. Abdominal trauma

Subject importance

| Null |  |  |  |  |  |  |  |  |  | Maximum |
| --- | --- | --- | --- | --- | --- | --- | --- | --- | --- | --- |
| 0 | 1 | 2 | 3 | 4 | 5 | 6 | 7 | 8 | 9 | 10 |

1. Massive haemorrhage management

Subject importance

| Null |  |  |  |  |  |  |  |  |  | Maximum |
| --- | --- | --- | --- | --- | --- | --- | --- | --- | --- | --- |
| 0 | 1 | 2 | 3 | 4 | 5 | 6 | 7 | 8 | 9 | 10 |

1. Trauma in the pregnant woman

Subject importance

| Null |  |  |  |  |  |  |  |  |  | Maximum |
| --- | --- | --- | --- | --- | --- | --- | --- | --- | --- | --- |
| 0 | 1 | 2 | 3 | 4 | 5 | 6 | 7 | 8 | 9 | 10 |

1. Simulation training on trauma - clinical cases

52.1 Subject importance

| Null |  |  |  |  |  |  |  |  |  | Maximum |
| --- | --- | --- | --- | --- | --- | --- | --- | --- | --- | --- |
| 0 | 1 | 2 | 3 | 4 | 5 | 6 | 7 | 8 | 9 | 10 |

52.2 Formative impact

| Null |  |  |  |  |  |  |  |  |  | Maximum |
| --- | --- | --- | --- | --- | --- | --- | --- | --- | --- | --- |
| 0 | 1 | 2 | 3 | 4 | 5 | 6 | 7 | 8 | 9 | 10 |

1. Pathophysiology and management of Acute Respiratory Distress Syndrome (ARDS)

Subject importance

| Null |  |  |  |  |  |  |  |  |  | Maximum |
| --- | --- | --- | --- | --- | --- | --- | --- | --- | --- | --- |
| 0 | 1 | 2 | 3 | 4 | 5 | 6 | 7 | 8 | 9 | 10 |

1. ARDS ventilation

Subject importance

| Null |  |  |  |  |  |  |  |  |  | Maximum |
| --- | --- | --- | --- | --- | --- | --- | --- | --- | --- | --- |
| 0 | 1 | 2 | 3 | 4 | 5 | 6 | 7 | 8 | 9 | 10 |

1. Pathophysiology of sepsis

Subject importance

| Null |  |  |  |  |  |  |  |  |  | Maximum |
| --- | --- | --- | --- | --- | --- | --- | --- | --- | --- | --- |
| 0 | 1 | 2 | 3 | 4 | 5 | 6 | 7 | 8 | 9 | 10 |

1. Management of a septic patient

Subject importance

| Null |  |  |  |  |  |  |  |  |  | Maximum |
| --- | --- | --- | --- | --- | --- | --- | --- | --- | --- | --- |
| 0 | 1 | 2 | 3 | 4 | 5 | 6 | 7 | 8 | 9 | 10 |

1. Simulation training on intensive care - clinical cases

57.1 Subject importance

| Null |  |  |  |  |  |  |  |  |  | Maximum |
| --- | --- | --- | --- | --- | --- | --- | --- | --- | --- | --- |
| 0 | 1 | 2 | 3 | 4 | 5 | 6 | 7 | 8 | 9 | 10 |

57.2 Formative impact

| Null |  |  |  |  |  |  |  |  |  | Maximum |
| --- | --- | --- | --- | --- | --- | --- | --- | --- | --- | --- |
| 0 | 1 | 2 | 3 | 4 | 5 | 6 | 7 | 8 | 9 | 10 |

1. Anatomo-physiological changes of pregnancy

Subject importance

| Null |  |  |  |  |  |  |  |  |  | Maximum |
| --- | --- | --- | --- | --- | --- | --- | --- | --- | --- | --- |
| 0 | 1 | 2 | 3 | 4 | 5 | 6 | 7 | 8 | 9 | 10 |

1. Labour analgesia

Subject importance

| Null |  |  |  |  |  |  |  |  |  | Maximum |
| --- | --- | --- | --- | --- | --- | --- | --- | --- | --- | --- |
| 0 | 1 | 2 | 3 | 4 | 5 | 6 | 7 | 8 | 9 | 10 |

1. Obstetric Emergencies

Subject importance

| Null |  |  |  |  |  |  |  |  |  | Maximum |
| --- | --- | --- | --- | --- | --- | --- | --- | --- | --- | --- |
| 0 | 1 | 2 | 3 | 4 | 5 | 6 | 7 | 8 | 9 | 10 |

1. Simulation training on obstetric anaesthesiology - clinical cases

61.1 Subject importance

| Null |  |  |  |  |  |  |  |  |  | Maximum |
| --- | --- | --- | --- | --- | --- | --- | --- | --- | --- | --- |
| 0 | 1 | 2 | 3 | 4 | 5 | 6 | 7 | 8 | 9 | 10 |

61.2 Formative impact

| Null |  |  |  |  |  |  |  |  |  | Maximum |
| --- | --- | --- | --- | --- | --- | --- | --- | --- | --- | --- |
| 0 | 1 | 2 | 3 | 4 | 5 | 6 | 7 | 8 | 9 | 10 |

1. Global Evaluation

| Null |  |  |  |  |  |  |  |  |  | Maximum |
| --- | --- | --- | --- | --- | --- | --- | --- | --- | --- | --- |
| 0 | 1 | 2 | 3 | 4 | 5 | 6 | 7 | 8 | 9 | 10 |


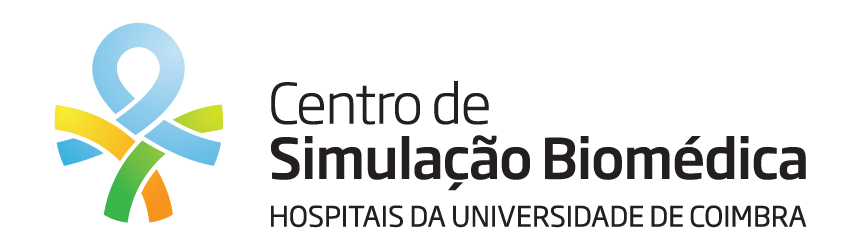
Choose a letter:

| **A** | **B** | **C** | **D** | **E** | **F** | **G** | **H** | **I** | **J** | **L** | **M** | **N** | **O** | **P** | **Q** | **R** | **S** | **T** |
| --- | --- | --- | --- | --- | --- | --- | --- | --- | --- | --- | --- | --- | --- | --- | --- | --- | --- | --- |

Questionnaire – Module IV

Dear colleague,

This questionnaire intends to assess the evolution of the participant during the training with simulation. The expected average time for completion is five minutes.

All data is confidential and used for research purposes only.

The medical team of Coimbra’s Biomedical Simulation Center be grateful for your cooperation.

Their fill shall be understood as an authorization to the assumptions referred above.

1. How do you assess your preparation for critical events in the operating room or emergency room?

| Null |  |  |  |  |  |  |  |  |  | Maximum |
| --- | --- | --- | --- | --- | --- | --- | --- | --- | --- | --- |
| 0 | 1 | 2 | 3 | 4 | 5 | 6 | 7 | 8 | 9 | 10 |

1. In your opinion, how important is…

… airway managment?

| Null |  |  |  |  |  |  |  |  |  | Maximum |
| --- | --- | --- | --- | --- | --- | --- | --- | --- | --- | --- |
| 0 | 1 | 2 | 3 | 4 | 5 | 6 | 7 | 8 | 9 | 10 |

2.1... ventilatory monitoring?

| Null |  |  |  |  |  |  |  |  |  | Maximum |
| --- | --- | --- | --- | --- | --- | --- | --- | --- | --- | --- |
| 0 | 1 | 2 | 3 | 4 | 5 | 6 | 7 | 8 | 9 | 10 |

2.2... cardiac monitoring?

| Null |  |  |  |  |  |  |  |  |  | Maximum |
| --- | --- | --- | --- | --- | --- | --- | --- | --- | --- | --- |
| 0 | 1 | 2 | 3 | 4 | 5 | 6 | 7 | 8 | 9 | 10 |

2.3… neuromuscular block monitoring?

| Null |  |  |  |  |  |  |  |  |  | Maximum |
| --- | --- | --- | --- | --- | --- | --- | --- | --- | --- | --- |
| 0 | 1 | 2 | 3 | 4 | 5 | 6 | 7 | 8 | 9 | 10 |

1. How do you evaluate your training ...

3.1… in difficult airway management?

| Null |  |  |  |  |  |  |  |  |  | Maximum |
| --- | --- | --- | --- | --- | --- | --- | --- | --- | --- | --- |
| 0 | 1 | 2 | 3 | 4 | 5 | 6 | 7 | 8 | 9 | 10 |

3.2… in advanced life support?

| Null |  |  |  |  |  |  |  |  |  | Maximum |
| --- | --- | --- | --- | --- | --- | --- | --- | --- | --- | --- |
| 0 | 1 | 2 | 3 | 4 | 5 | 6 | 7 | 8 | 9 | 10 |

3.3… for emergencies in your clinical practice?

| Null |  |  |  |  |  |  |  |  |  | Maximum |
| --- | --- | --- | --- | --- | --- | --- | --- | --- | --- | --- |
| 0 | 1 | 2 | 3 | 4 | 5 | 6 | 7 | 8 | 9 | 10 |

3.4… in crisis resource management?

| Null |  |  |  |  |  |  |  |  |  | Maximum |
| --- | --- | --- | --- | --- | --- | --- | --- | --- | --- | --- |
| 0 | 1 | 2 | 3 | 4 | 5 | 6 | 7 | 8 | 9 | 10 |

1. How do you rate your expertise…

4.1… for difficult airway management?

| Null |  |  |  |  |  |  |  |  |  | Maximum |
| --- | --- | --- | --- | --- | --- | --- | --- | --- | --- | --- |
| 0 | 1 | 2 | 3 | 4 | 5 | 6 | 7 | 8 | 9 | 10 |

4.2… in advanced life support?

| Null |  |  |  |  |  |  |  |  |  | Maximum |
| --- | --- | --- | --- | --- | --- | --- | --- | --- | --- | --- |
| 0 | 1 | 2 | 3 | 4 | 5 | 6 | 7 | 8 | 9 | 10 |

4.3… for emergencies in your clinical practice?

| Null |  |  |  |  |  |  |  |  |  | Maximum |
| --- | --- | --- | --- | --- | --- | --- | --- | --- | --- | --- |
| 0 | 1 | 2 | 3 | 4 | 5 | 6 | 7 | 8 | 9 | 10 |

4.4… in crisis resource management?

| Null |  |  |  |  |  |  |  |  |  | Maximum |
| --- | --- | --- | --- | --- | --- | --- | --- | --- | --- | --- |
| 0 | 1 | 2 | 3 | 4 | 5 | 6 | 7 | 8 | 9 | 10 |

1. I've been in situations that I couldn’t deal without help.

| Never | Few times | Many times |
| --- | --- | --- |
| 🞎 | 🞎 | 🞎 |

1. I call for help

| Never | Few times | Many times |
| --- | --- | --- |
| 🞎 | 🞎 | 🞎 |

1. I feel the need for support

| Never | Few times | Many times |
| --- | --- | --- |
| 🞎 | 🞎 | 🞎 |

1. I make mistakes

| Never | Few times | Many times |
| --- | --- | --- |
| 🞎 | 🞎 | 🞎 |

1. It’s difficult for me to report the mistakes I make

| Strongly disagree | Partially disagree | No opinion | Partially agree | Strongly agree |
| --- | --- | --- | --- | --- |
| 🞎 | 🞎 | 🞎 | 🞎 | 🞎 |

1. I don’t feel prepared for the responsibility I have

| Strongly disagree | Partially disagree | No opinion | Partially agree | Strongly agree |
| --- | --- | --- | --- | --- |
| 🞎 | 🞎 | 🞎 | 🞎 | 🞎 |

1. I don’t have enough knowledge for the responsibility I have

| Strongly disagree | Partially disagree | No opinion | Partially agree | Strongly agree |
| --- | --- | --- | --- | --- |
| 🞎 | 🞎 | 🞎 | 🞎 | 🞎 |

1. I don’t have enough training for the responsibility I have

| Strongly disagree | Partially disagree | No opinion | Partially agree | Strongly agree |
| --- | --- | --- | --- | --- |
| 🞎 | 🞎 | 🞎 | 🞎 | 🞎 |

1. I don’t have enough experience for the responsibility I have

| Strongly disagree | Partially disagree | No opinion | Partially agree | Strongly agree |
| --- | --- | --- | --- | --- |
| 🞎 | 🞎 | 🞎 | 🞎 | 🞎 |

1. I feel bad when I call for help

| Strongly disagree | Partially disagree | No opinion | Partially agree | Strongly agree |
| --- | --- | --- | --- | --- |
| 🞎 | 🞎 | 🞎 | 🞎 | 🞎 |

1. When I disagree with the consultant anesthesiologist opinion, I don’t express that position.

| Strongly disagree | Partially disagree | No opinion | Partially agree | Strongly agree |
| --- | --- | --- | --- | --- |
| 🞎 | 🞎 | 🞎 | 🞎 | 🞎 |

1. The behavioural component is crucial in the clinical setting

| Strongly disagree | Partially disagree | No opinion | Partially agree | Strongly agree |
| --- | --- | --- | --- | --- |
| 🞎 | 🞎 | 🞎 | 🞎 | 🞎 |

1. Simulation team training is an important complement to the residency program

| Strongly disagree | Partially disagree | No opinion | Partially agree | Strongly agree |
| --- | --- | --- | --- | --- |
| 🞎 | 🞎 | 🞎 | 🞎 | 🞎 |

1. A regular simulation update plan should be defined

| Strongly disagree | Partially disagree | No opinion | Partially agree | Strongly agree |
| --- | --- | --- | --- | --- |
| 🞎 | 🞎 | 🞎 | 🞎 | 🞎 |

1. Simulation team training improves clinical daily practice

| Strongly disagree | Partially disagree | No opinion | Partially agree | Strongly agree |
| --- | --- | --- | --- | --- |
| 🞎 | 🞎 | 🞎 | 🞎 | 🞎 |

1. Simulation team training have an impact on patients' clinical outcome

| Strongly disagree | Partially disagree | No opinion | Partially agree | Strongly agree |
| --- | --- | --- | --- | --- |
| 🞎 | 🞎 | 🞎 | 🞎 | 🞎 |

**Course evaluation**

**Pedagogical content**

1. Effective communication

Subject importance

| Null |  |  |  |  |  |  |  |  |  | Maximum |
| --- | --- | --- | --- | --- | --- | --- | --- | --- | --- | --- |
| 0 | 1 | 2 | 3 | 4 | 5 | 6 | 7 | 8 | 9 | 10 |

1. Crisis Resource Management in Anaesthesiology (ACRM)

Subject importance

| Null |  |  |  |  |  |  |  |  |  | Maximum |
| --- | --- | --- | --- | --- | --- | --- | --- | --- | --- | --- |
| 0 | 1 | 2 | 3 | 4 | 5 | 6 | 7 | 8 | 9 | 10 |

1. ACRM principle

Subject importance

| Null |  |  |  |  |  |  |  |  |  | Maximum |
| --- | --- | --- | --- | --- | --- | --- | --- | --- | --- | --- |
| 0 | 1 | 2 | 3 | 4 | 5 | 6 | 7 | 8 | 9 | 10 |

1. Simulation training on ACRM - clinical cases

66.1 Subject importance

| Null |  |  |  |  |  |  |  |  |  | Maximum |
| --- | --- | --- | --- | --- | --- | --- | --- | --- | --- | --- |
| 0 | 1 | 2 | 3 | 4 | 5 | 6 | 7 | 8 | 9 | 10 |

66.2 Formative impact

| Null |  |  |  |  |  |  |  |  |  | Maximum |
| --- | --- | --- | --- | --- | --- | --- | --- | --- | --- | --- |
| 0 | 1 | 2 | 3 | 4 | 5 | 6 | 7 | 8 | 9 | 10 |

1. Emergencies in the Operating room

Subject importance

| Null |  |  |  |  |  |  |  |  |  | Maximum |
| --- | --- | --- | --- | --- | --- | --- | --- | --- | --- | --- |
| 0 | 1 | 2 | 3 | 4 | 5 | 6 | 7 | 8 | 9 | 10 |

1. Simulation training operating room emergencies: clinical cases

68.1 Subject importance

| Null |  |  |  |  |  |  |  |  | Maximum |
| --- | --- | --- | --- | --- | --- | --- | --- | --- | --- |
| 0 | 2 | 3 | 4 | 5 | 6 | 7 | 8 | 9 | 10 |

68.2 Formative impact

| Null |  |  |  |  |  |  |  |  |  | Maximum |
| --- | --- | --- | --- | --- | --- | --- | --- | --- | --- | --- |
| 0 | 1 | 2 | 3 | 4 | 5 | 6 | 7 | 8 | 9 | 10 |

1. Global evaluation

| Null |  |  |  |  |  |  |  |  |  | Maximum |
| --- | --- | --- | --- | --- | --- | --- | --- | --- | --- | --- |
| 0 | 1 | 2 | 3 | 4 | 5 | 6 | 7 | 8 | 9 | 10 |
